# Phylogeography and genetic structure of the oriental river prawn *Macrobrachium nipponense* (Crustacea: Decapoda: Palaemonidae) in East Asia

**DOI:** 10.1371/journal.pone.0173490

**Published:** 2017-03-07

**Authors:** Po-Cheng Chen, Chun-Han Shih, Ta-Jen Chu, Ying-Chou Lee, Tzong-Der Tzeng

**Affiliations:** 1 Institute of Fisheries Science, College of Life Science, National Taiwan University, Taipei, Taiwan; 2 Department of Leisure Management, Tungnan University, New Taipei City, Taiwan; 3 Department of Leisure and Recreation Management, Chung Hua University, Hsin Chu, Taiwan; 4 Department of Leisure, Recreation and Tourism Management, Shu-Te University, Kaohsiung, Taiwan; National Cheng Kung University, TAIWAN

## Abstract

The oriental river prawn (*Macrobrachium nipponense*) is mainly distributed in East Asia. The phylogeography, population genetic structure and historical demography of this species in the East Asia were examined by using partial sequences of the cytochrome oxidase subunit I (COI) and 16S rRNA in mitochondrial DNA. Ten populations that included 239 individuals were collected from Taiwan (Shihmen Reservoir, SMR, Mingte Reservoir, MTR and Chengching Lake Reservoir, CLR), mainland China (Taihu Lake, TLC, Min River, MRC, Jiulong River, JRC and Shenzhen Reservoir, SRC), Japan (Biwa Lake, BLJ and Kasumigaura Lake, KLJ) and Korea (Han River, HRK). The nucleotide diversity (*π*) of all individuals was 0.01134, with values ranging from 0.0089 (BLJ, Japan) to 0.01425 (MTR, Taiwan). A total of 83 haplotypes were obtained, and the haplotypes were divided into 2 main lineages: lineage A included the specimens from BLJ, KLJ, CLR, MTR, TLC, MRC and JRC, and lineage B comprised the ones from HRK, SRC, SMR, MTR, TLC, MRC and JRC. Lineage A could be further divided two sub-lineages (A_1_ and A_2_). Individuals of lineage A_2_ were only from TLC. Demographic expansion was observed in each lineage, starting within the second-to-latest interglacial period for lineage A and within the last glacial period for lineage B. All *F*_ST_ values among the ten populations were significantly different, except for the values between MRC and JRC, and SMR and SRC. The phylogeography and genetic structure of *M*. *nipponense* in East Asia might be influenced by Pleistocene glacial cycles, lake isolation and human introduction. The possible dispersal routes of *M*. *nipponense* in the East Asia were also discussed.

## Introduction

The complex geological events and climatic history of various regions helped shape current phylogeographical patterns [1, 2]. Therefore, the current population genetic structure of a specific species had been influenced by the interactions of biology, geography, and climatic shifts [3]. During the last glacial maximum, the sea level was 130–150 m lower than the present level in the East China Sea and 100–120 m lower in the South China Sea [4, 5]. Consequently, the entire Bohai gulf, the Yellow Sea, and the Tsushima and Taiwan Straits were exposed, and the islands of Taiwan, Japan and Korea were linked to mainland China [6]. Moreover, recent studies have shown that there are many rivers that were separated by the sea but became connected to one another during the glacial period, and this resulted in gene flow between different river systems [7]. Therefore, the present genetic structures of populations in the marginal seas of the East Asia have been greatly affected by ice ages.

The gene flow of inland freshwater between populations is obviously lower than the one between populations in estuaries or oceans, and thus historical phylogeographical analyses of freshwater species permit strong inferences regarding the biotic and geological evolution of a region [8, 9]. More recently, the climatic change can create great changes in species’ geographical distributions and biotic richness from some theoretical and empirical studies, and the advent of DNA technology provides proper markers to reflect the genetic effects of adaptation as well. [2, 3]. Mitochondrial (mt) DNA has many attributes that make it particularly suitable for population genetic studies, including its rapid rate of evolution, a lack of recombination, and its maternal inheritance [10, 11].

The oriental river prawn (*Macrobrachium nipponense*) originated in mainland China about one million years ago [12], and is mainly distributed over East Asian regions including mainland China, Japan, Korea, and Taiwan. This species has the potential for aquaculture because it can reproduce easily and is highly tolerant of various environments [13]. In fact, *M*. *nipponense* is considered as one of the most important freshwater prawns for aquaculture in China [14].

Many studies on the population structure of the oriental river prawn were conducted in East Asia, but the populations analyzed in those papers were collected from one specific country [15]. One of the newest paper using mitochondrial DNA sequences to determine the population structure of this species was performed in Taiwan [16], and two different lineages were found. Various DNA markers or techniques including RAPD, ISSR and COI gene were applied to determine the population structure of this species in China [17, 18, 19, 20, 21, 22]. Two different lineages were also found. Different genetic markers were also applied to elucidate the population genetic structure of this species in Japan [13, 14, 23], and different clades were found. However, the phylogeography and the population genetic structure of *M*. *nipponense* from different area in East Asia is still unknown. In this study, we used mtDNA fragment sequences of cytochrome oxidase subunit I (COI) and the 16S rRNA gene to reveal the phylogeography, the population genetic structure and historical demography of the oriental river prawn populations in East Asia.

## Materials and methods

### Sample collection

**Fig 1:**
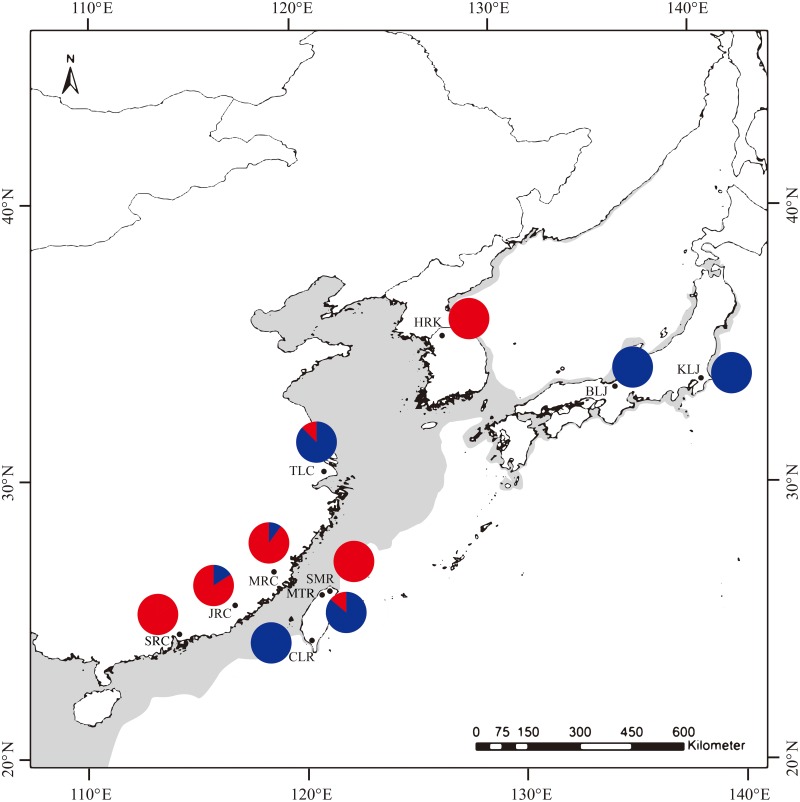
Sampling localities and haplotypes frequencies of *Macrobrachium nipponense* in Taiwan. Numbers of lineages A and B in each sampling site are also shown in Table 1.

All samples were collected in open and public waters, and thus specific permission is not necessary. The specimens were collected by five bait traps each reservoir and each estuary from the evening 17:00 to next morning 7:00 during successive 3 days in winter season, from December 2013 to March 2014. Ten populations that included 239 individuals from Taiwan (Shihmen Reservoir, SMR, Mingte Reservoir, MTR and Chengching Lake Reservoir, CLR), mainland China (Taihu Lake, TLC, Min River, MRC, Jiulong River, JRC and Shenzhen Reservoir, SRC), Japan (Biwa Lake, BLJ and Kasumigaura Lake, KLJ) and Korea (Han River, HRK) were collected respectively (Fig 1; Table 1). The specimens were immediately iced or frozen after capture and kept at -75°C for DNA extraction.

**Table 1:** Codes of sampling sites, sample sizes (*n*), number of haplotypes (*n*_*h*_), gene diversity (*h*), nucleotide diversity (*π*), Tajimatid*D*, and Fuati*Fs* statistics for 10 populations of *Macrobrachium nipponense* in East Asia.

Code	Sample site	*n*	*n*_*h*_	Lineage A	Lineage B	All populations		Lineage A			Lineage B			Tajima's D	Fu's Fs
						*h*	π	*n*_*h*_	*h*^a^	π^a^	*n*_*h*_	*h*^a^	π^a^		
SMR	Shihmen Reservoir	26	6	0	26	0.658 ± 0.062	0.002 ± 0.001	-	-	-	6	-	0.002 ± 0.001	-1.409	-0.675
MTR	Mingte Reservoir	24	18	19	5	0.964 ± 0.025	0.014 ± 0.003	13	0.942 ± 0.037	0.013 ± 0.001	-	-	-	-1.296	-3.583*
CLR	Chengching Lake Reservoir	22	11	22	0	0.883 ± 0.053	0.007 ± 0.002	11	0.883 ± 0.053	0.007 ± 0.002	-	-	-	-1.794	-1.103
BLJ	Biwa Lake, Japan	30	4	30	0	0.572 ± 0.052	0.001 ± 0.000	4	0.572 ± 0.052	0.001 ± 0.000	-	-	-	-0.767	-0.417
KLJ	Kasumigaura Lake, Japan	20	3	20	0	0.695 ± 0.040	0.002 ± 0.000	3	0.695 ± 0.040	0.002 ± 0.000	-	-	-	1.646	2.797
TLC	Taihu Lake, China	24	15	21	3	0.931 ± 0.034	0.008 ± 0.001	14	0.924 ± 0.043	0.006 ± 0.001	-	-	-	-1.248	-3.509*
MRC	Min River, China	25	9	2	23	0.683 ± 0.100	0.004 ± 0.001	-	-	-	7	0.625 ± 0.110	0.002 ± 0.001	-1.400	-1.241
JRC	Jiulong River, China	22	10	4	18	0.896 ± 0.042	0.007 ± 0.001	-	-	-	8	0.856 ± 0.059	0.003 ± 0.001	0.156	-0.430
SRC	Shenzhen Reservoir, China	15	8	0	15	0.867 ± 0.067	0.009 ± 0.004	-	-	-	8	0.867 ± 0.067	0.009 ± 0.004	-2.202**	0.678
HRK	Han River, Korea	31	9	0	31	0.692 ± 0.088	0.002 ± 0.001	-	-	-	9	0.692 ± 0.088	0.002 ± 0.001	-1.781	-2.960*
Lineage A		118	47						0.956 ± 0.008	0.009 ± 0.001				-1.904*	-22.751**
Lineage A_1_		101	37						0.943 ± 0.011	0.008 ± 0.001				-1.908*	-15.057**
Lineage A_2_		17	10						0.924 ± 0.043	0.006 ± 0.001				-1.766	-3.571**
Lineage B		121	31									0.866 ± 0.022	0.005 ± 0.001	-2.384**	-19.515**
Total		239	83			0.956 ± 0.007	0.011 ± 0.001							-2.110*	-50.334**

* *P* < 0.05,

** *P* < 0.01.

^a^ The number of sample size less than 5 for lineage A or B not calculated.

### DNA extraction, amplification and sequencing

Total genomic DNA was extracted from pereopod muscle using QIAamp DNA Mini Kit.[24]. Two different fragments (16S rRNA and COI) of mtDNA were amplified and sequenced. The 16S rRNA and COI sequences were amplified using 1471 (5’-CCT GTT TAN CAA AAA CAT-3’) and 1472 (5’-AGA TAG AAA CCA ACC TGG-3’) [25], and COI-F (TTT ATC TTC GGA GCG TGA GC) and COI-R (AGT TAT TCC TGG GGC TCG TAT G) [26] primers, respectively. Thermal cycling was performed on a GeneAmp 2400 thermal cycler (Perkin-Elmer, Norwalk, CT, USA), and PCR conditions consisted of 39 cycles of denaturation at 95°C for 50 s, annealing at 50°C for 1 min, and extension at 72°C for 1.5 min. An initial denaturation step at 95°C for 5 min and a final extension holding at 72°C for 10 min were included in the 1st and last cycles, respectively. The PCR product was separated by electrophoresis on 1.5% agarose gels, purified with the Gene Clean II kit (Bio101, Vista, CA, USA), and sequenced on an ABI 377 DNA sequencer (Applied Biosystems, Inc.; Foster City, CA, USA).

### Sequence analyses

All sequences were aligned using MegAlign (DNASTAR, LaserGene, WI, USA). The number of variable and parsimony informative sites, base composition, haplotype diversity and nucleotide diversity [27] were calculated using DnaSP version 5.00 [28].

Part of the sequences of the 16S rRNA and COI genes were concatenated in the following analyses. Phylogeographic analyses of 16S rRNA and COI genes were carried out by the neighbour-joining (NJ) and maximum likelihood (ML) methods, respectively, by MEGA 6 [16, 29]. Bootstrap analyses with 1,000 replicates were used to evaluate the phylogenetic relationships of all haplotypes. The optimal substitution model was determined using MEGA. A network of haplotypes was also constructed using the median-joining method [30] in Network version 4.6.1.3, available at http://www.fluxus-engineering.com. The historical demographic expansion was investigated by examining the frequency distributions of pair-wise differences between sequences (mismatched distribution) with ARLEQUIN [16]. The approximate age of the population or lineage was estimated with the formula A = μπ [31]. A is the age of the population or lineage, *π* is nucleotide diversity and μ is μ (the mutation rate) x generation time; the approximate dates of population expansion were estimated with the formula τ = 2μ*T* [32], where *T* is the time since expansion, τ is the expansion time, and 2μ is μ (the mutation rate) x generation time x the number of bases sequenced. The average divergence rate of 1.17–1.66% per million years and a generation time of one year were used [33].

To examine the genetic differentiation between any two populations, pair-wise *F*_ST_ statistics were estimated by ARLEQUIN version 3.5 [34]. A dendrogram of the ten sampling sites was also constructed using the unweighted pair-group method with arithmetic means (UPGMA) based on the *F*_ST_ values. The population structure was also assessed by an analysis of molecular variance (AMOVA; [35] in ARLEQUIN). Various groupings of these populations were suggested by an UPGMA tree of these ten populations. The grouping that revealed the maximal value of Φ_CT_ and significantly differed from a random organization of similar groupings was assumed to represent the most-probable geographic subdivisions [36]. The significance test of the statistical result was evaluated by a permutations test with 10,000 random permutations.

Tajima’s *D* [37] was used to check for deviations from neutrality, indicating whether population expansion had occurred in the past. Fu’s *Fs* test [38] was also carried out to assess the evidence for population expansion using DnaSP. In addition, population expansion was also investigated with a mismatch analysis to examine the frequency distributions of the nucleotide difference as a function of frequency using DnaSP.

The Mantel test [39], available in ARLEQUIN, was used to test for isolation by distance. We used the pair-wise *F*_ST_ values and the corresponding pair-wise geographical distances as the input data, and 1000 permutations were performed to determine the level of significance. The approximate geographic distances between sampling locations were used as the minimum distance map.

## Results

In total, 239 specimens were sequenced. The size of the 16S rRNA fragment was 421 bp, with 78 variable sites and 37 parsimony informative sites, resulting in 38 unique haplotypes (S1 Dataset). All sequences were deposited in GenBank, with accession numbers KU235597—KU235646, KU235699—KU235720 and KY084569—KY084735. No gap was detected. The frequency of the nucleotide composition showed an AT bias (with G + C contents of 33.7%). The size of the COI fragment was 371 bp; there were 42 variable sites and 31 parsimony informative sites, resulting in 47 unique haplotypes (S2 Dataset). All sequences were deposited in GenBank, with accession numbers KU235799—KU235848, KU235901—KU235922 and KY092174—KY092340. The frequency of nucleotide composition showed an AT bias (with G + C contents of 39.8%).

The following results were obtained by analyzing the combined sequences of the 16S rRNA and COI genes (S3 Dataset). The haplotype diversity (*h*) of all ten populations was 0.956, with values that ranged from 0.572 (BLJ) to 0.964 (MTR) (Table 1). The nucleotide diversity (*π*) of all populations was 0.011, with values that ranged from 0.001 (BLJ) to 0.014 (MTR) (Table 1). A total of 83 haplotypes was detected in 239 specimens. The most common allele was shared by 36 individuals from the HRK (17), SMR (10), MRC (3), JRC (3) and SRC (3) populations. The second most common allele was shared by 20 individuals from the populations of MRC (14) and JRC (6). The third most common alleles were shared by 17 individuals from the populations of SMR (12) and SRC (5).

**Fig 2:**
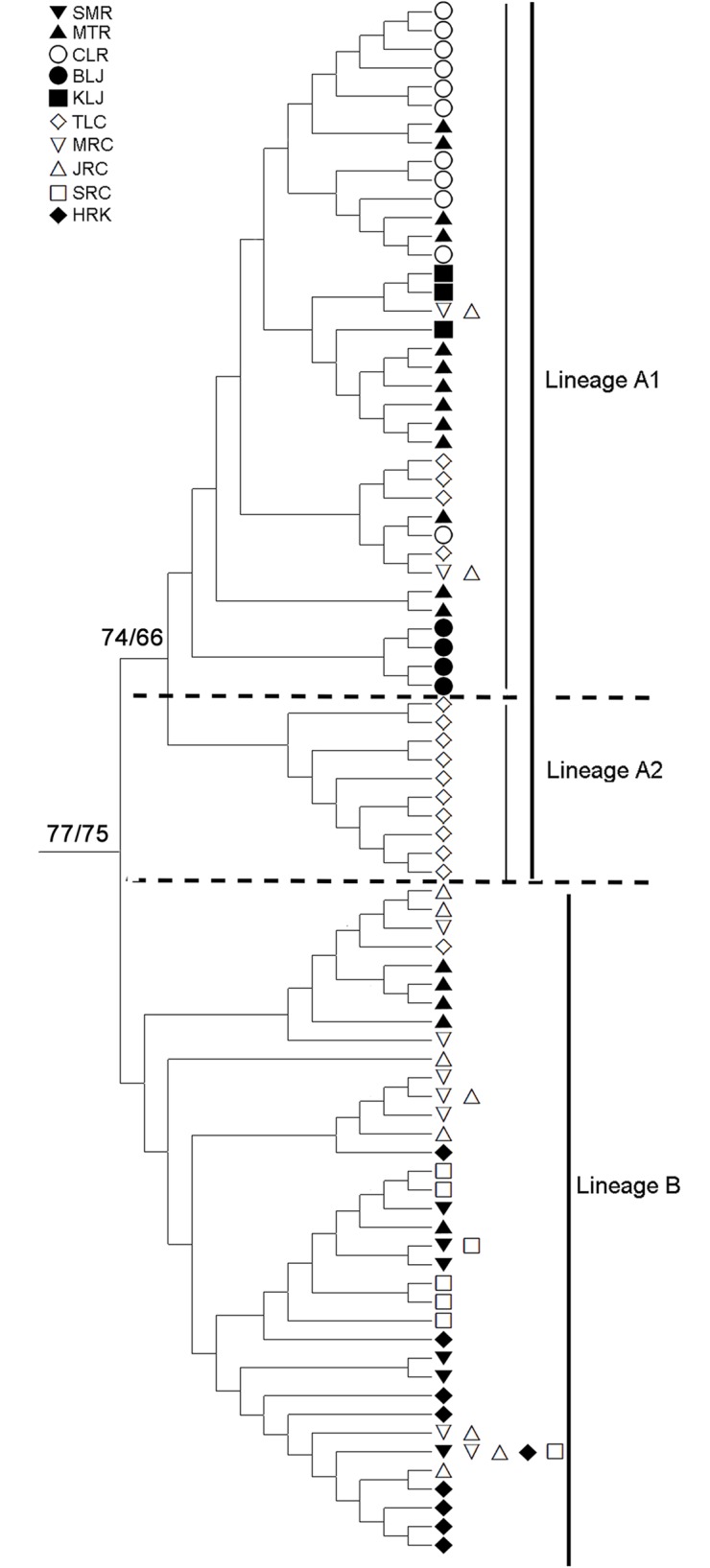
Neighbour-Joining (NJ) tree based on mtDNA 16S rRNA and COI sequences with bootstrap values (NJ/ML, respectively) shown adjacent to the corresponding two lineages for *Macrobrachium nipponense*. The numbers at the nodes indicate bootstrap values (expressed as percentage) with 1,000 replicates.

**Fig 3:**
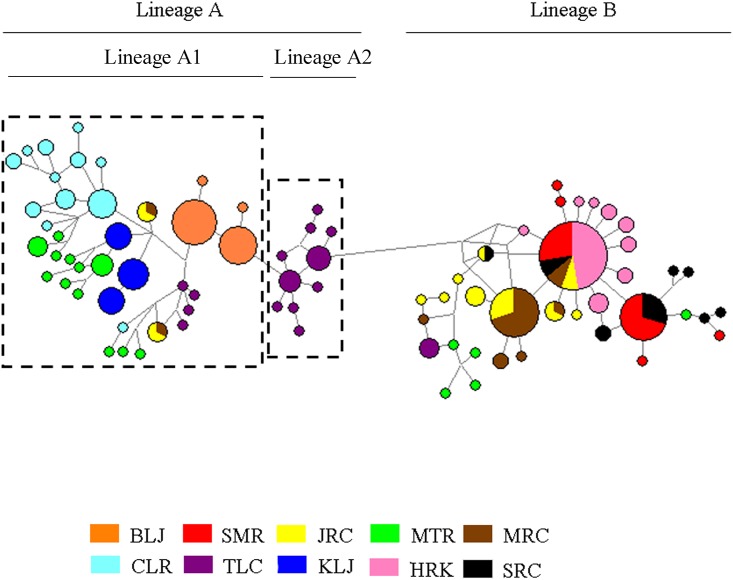
The haplotype network of *Macrobrachium nipponense* in all sampling sites.

The best-fitting model explaining our data was the K2 model. This model was used for NJ and ML reconstructions and AMOVA analyses. A phylogenetic tree of all haplotypes is shown in Fig 2. The results of both the NJ and ML trees were very similar. Two distinct lineages (A and B) were found. Lineage A might be further divided into two sub-lineages (A_1_ and A_2_). Bootstrap values are 77 and 75 for the NJ and ML trees between lineage A and lineage B, respectively. The bootstrap values are 74 and 66 for the NJ and ML trees between lineage A_1_ and lineage A_2_, respectively. The network for all specimens (Fig 3) supported the result obtained from these phylogenetic trees. Two sub-lineages were also found in lineage A in the network. The distribution of specimen of lineages A and B for different populations are also shown in Fig 1 and Table 1. All individuals from CLR, KLJ and BLJ were only included in lineage A, and all individuals from SMR, SRC and HRK were only involved in lineage B. All specimen from the other four sites were distributed into the two lineages at the same time.

The haplotype diversities (h) of lineages A, A_1_, A_2_ and lineage B were 0.955, 0.943 0.882 and 0.866, respectively. The nucleotide diversities (π) of lineages A, A_1_, A_2_ and lineage B were 0.009, 0.008, 0.006 and 0.005, respectively (Table 1). The τ values of lineages A, A_1_, A_2_ and lineage B were 4.713/2μ, 4.237/2μ, 0.812/2μ, 1.108/2μ generations, respectively. The average mutation rate of 1.42% / myr and a generation time of 1 year were used to calculate the time of expansion. The estimated time of expansion for lineage A was 209,533 years ago, A_1_ was 188,371 years ago, and A_2_ was 36,100 years ago. For lineage B, the estimate was 49,248 years ago.

**Fig 4:**
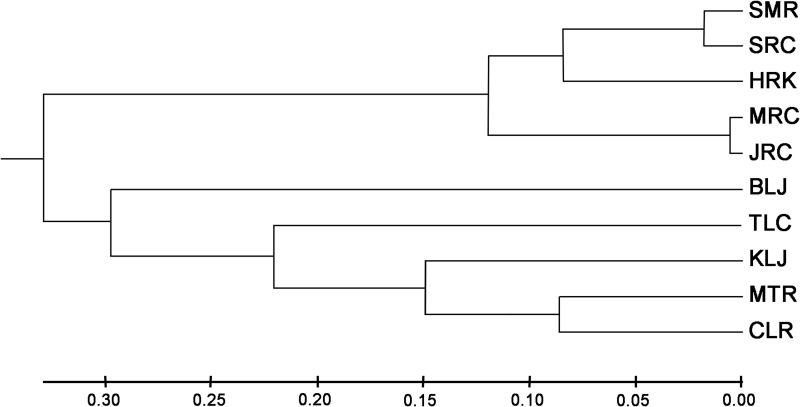
UPGMA tree showing relationships among the 10 sampling sites.

All *F*_ST_ values among the ten populations were significant, except for the ones between MRC and JRC and between SMR and SRC (Table 2). The UPGMA tree of these ten sampling areas could be divided into two main groups (Fig 4); the first group included the SMR, HRK, SRC, MRC and JRC, and the second group included the other five populations. The second group may be further divided into four subgroups; the first subgroup included the CLR and MTR populations; the second subgroup included the KLJ; the third subgroup included TLC; and the fourth subgroup included BLJ.

**Table 2:** Matrix of pairwise *F*_*ST*_ (below diagonal) and *P* values (above diagonal) among 10 populations of *Macrobrachium nipponense* in East Asia.

	SMR	MTR	CLR	BLJ	KLJ	TLC	MRC	JRC	SRC	HRK
SMR	-	0.000	0.000	0.000	0.000	0.000	0.000	0.000	**0.063**	0.000
MTR	0.553	-	0.000	0.000	0.000	0.000	0.000	0.000	0.000	0.000
CLR	0.781	0.172	-	0.000	0.000	0.000	0.000	0.000	0.000	0.000
BLJ	0.900	0.425	0.635	-	0.000	0.000	0.000	0.000	0.000	0.000
KLJ	0.882	0.227	0.369	0.776	-	0.000	0.000	0.000	0.000	0.000
TLC	0.592	0.297	0.473	0.549	0.553	-	0.000	0.000	0.000	0.000
MRC	0.297	0.477	0.687	0.806	0.791	0.455	-	**0.189**	0.000	0.000
JRC	0.265	0.380	0.584	0.699	0.678	0.352	**0.010**	-	0.000	0.000
SRC	**0.035**	0.434	0.663	0.787	0.757	0.460	0.222	0.194	-	0.000
HRK	0.170	0.590	0.784	0.888	0.874	0.599	0.231	0.222	0.167	-

Five different groupings for the ten populations were suggested by the UPGMA trees. The results of the AMOVA are shown in Table 3. The AMOVA for the ten populations yielded a significant *F*_ST_ value of 0.57445, indicating that at least one of the pair-wise populations had significant heterogeneity. Significant values of Φ_CT_ were observed in all groupings. The highest Φ_CT_ values (0.4817) were found in grouping 2, and supported the conclusion that these ten populations could be divided into two main groups: the first group included SMR, HRK, SRC, MRC, and JRC, and the second group included the other five populations. Significant Φ_CT_ values were also found in different groupings, indicating that an additional genetic discontinuity may also have occurred among populations.

**Table 3:** AMOVA results for 10 populations of *Macrobrachium nipponense* in Taiwan.

Population	Grouping	Source of variation	Percentage of variation	Φ-Statistics	p (more-extreme value)
One group	Group 1{SMR, MTR, CLR, BLJ, KLJ, TLC, MRC, JRC, SRC, HRK}	AP	57.45	Φ_ST_ = 0.5745	***
		WP	42.55		
Two groups	Group 1{MTR, CCL, BLJ, KLJ, TLC}	AG	48.17	Φ_CT_ = 0.4817	***
	Group 2 {SMR, HRK, SRC, MRC, JRC}	AP/WG	18.34	Φ_SC_ = 0.3539	***
		WP	33.49	Φ_ST_ = 0.6651	***
Three groups	Group 1 {CLR, MTR, KLJ, TLC}	AG	48.14	Φ_CT_ = 0.4814	***
	Group 2 {SMR, HRK, SRC, MRC, JRC}	AP/WG	16.21	Φ_SC_ = 0.3125	***
	Group 3 {BLJ}	WP	35.65	Φ_ST_ = 0.6435	***
Four groups	Group 1{CLR, MTR, TLC}	AG	47.98	Φ_CT_ = 0.4798	***
	Group 2 {SMR, HRK, SRC, MRC, JRC}	AP/WG	15.23	Φ_SC_ = 0.2928	***
	Group 3{BLJ}	WP	36.79	Φ_ST_ = 0.6321	***
	Group 4{KLJ}				
Five groups	Group 1{CCL, MTR}	AG	54.68	Φ_CT_ = 0.5468	***
	Group 2 {SMR, HRK, SRC, MRC, JRC}	AP/WG	8.33	Φ_SC_ = 0.1839	***
	Group 3{TLC}	WP	36.99	Φ_ST_ = 0.6301	***
	Group 4{BLJ}				
	Group 5{KLJ}				

AG is the among-group component of variance; AP/WG is the among-populations/within-group component of variance; and WP is the within-population component of variance.

*** *P* < 0.001 by the permutation test.

**Fig 5:**
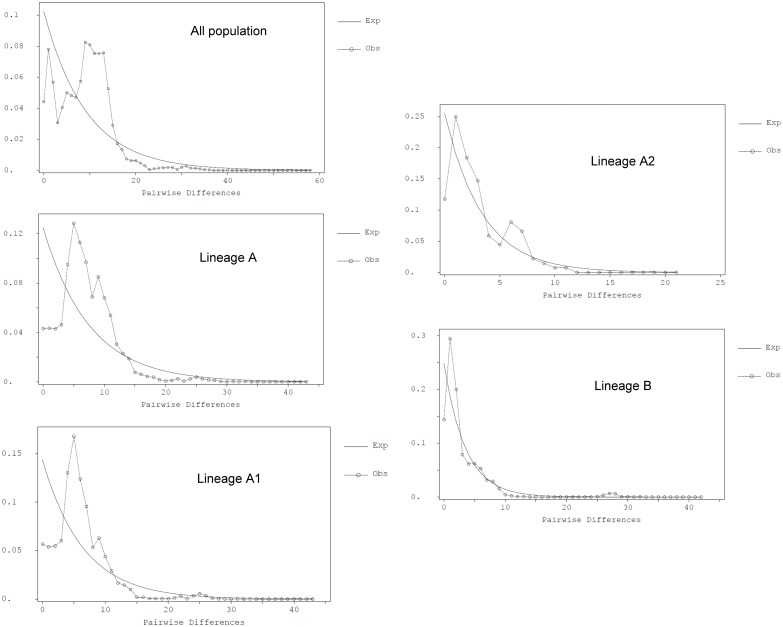
The observed pair-wise differences and the expected mismatch distributions under the sudden expansion model of oriental river prawn. (a) All populations, (b) Lineage A, (c) Lineage B.

No significant Tajima’s *D* values were found for all populations, except SRC (Table 1). However, Tajima’s *D* values were significant for each lineage and for the total population. Fu’s *F*s tests were significant for the MTR, TLC and HRK populations (Table 1). Significant Fu’s *F*s values were also obtained for lineages A and B and for the total population. The mismatched distribution of all specimens was bimodal (Fig 5a), with one mode corresponding to the number of differences within the lineages and the other mode corresponding to differences between the two lineages. A unimodal distribution was obtained from either lineage A or B, which did not significantly differ (as measured by the sum of the squared deviation; *P* > 0.05) from that predicted by the growth expansion model (Fig 5b and 5e). A unimodal distribution was also obtained from either lineage A_1_ or A_2_ (Fig 5c and 5d).

No correlations between genetic differentiation and the distance of geographic separation among populations were observed for lineages A and B (lineage A: *p* = 0.838; lineage B: *p* = 0.835), which indicates that oriental river prawns did not conform to an isolation-by-distance model of maternal gene flow.

## Discussion

Two distinct lineages (A and B) of *M*. *nipponense* in East Asia were found in this paper, and this result is the same as the one obtained in our previous paper [16]. Populations with ancestral genotypes tend to preserve higher nucleotide and haplotype diversities because of the long-term accumulation of mutations [40, 41, 42]. The nucleotide diversity (π = 0.009) and haplotype diversity (h = 0.956) in lineage A were significantly higher than the values (π = 0.005; h = 0.866) in lineage B. This suggested that lineage A was older than lineage B. The approximate age of lineages A and B calculated were 490,000 and 253,000 years, respectively (the average mutation rate of 1.42% / myr was used to calculate the age of the population or lineage). The estimate of time of expansion for lineage A (209,533 yrs ago), lineage A_1_ (188,371 yrs ago), lineage A_2_ (36,100 yrs ago) and lineage B (49,248 yrs ago) also proved this result.

Mismatch distributions of the two distinct lineages show that lineage B had a steeper peak, which indicates that there is a smaller original population before an expansion or bottleneck (Fig 5) [32]. This picture also suggests that lineage B could have experienced expansion in the more-recent past than lineage A, the pairwise distribution mode of which was more-clearly displaced to the right of the distribution pattern (Fig 5). This supported the conclusion that the time of expansion of lineage A was earlier than that of lineage B.

The oriental river prawn originated in mainland China [12], and thus, the high genetic diversity of populations in mainland China were expected. However, the nucleotide diversities (0.014) and haplotype diversity (0.964) in the MTR were significant higher than the values in these sites in mainland China and that may partly result from the specimens in MTR that were involved in two different lineages (Figs 1, 2 and 3). However, the estimated nucleotide diversity (0.013) and haplotype diversity (0.942) (Table 1) excluding the specimens that belonged to lineage B in MTR were still significantly higher than the values in different populations (such as TLC, π = 0.006, h = 0.924) (Table 1). Most individuals in MTR formed various unique haplotypes, not shared by other individuals from other sampling sites (Fig 3). This indicated that the MTR population had a longer time to accumulate mutations than the individuals from different sampling sites. Moreover, the haplotypes from the MTR population were distributed in two different locations in sub-lineage A_1_ (Figs 2 and 3), and this revealed that two different ancestral groups were recruited in the MTR population. Thus, these two different recruited ancestral groups may also cause the high diversity in the MTR population. We found that the haplotypes from the TLC population were also divided into two different sub-lineages (A_1_ and A_2_) (Figs 2 and 3), and this might reveal secondary back contact between the TLC and Japan populations occurred in this sampling site. However, the nucleotide diversities (0.008) and haplotype diversity (0.931) of the TLC population were still significantly lower than the values obtained from MTR. A large number of cultured prawns released into TLC in recent years [29] may have led to lower genetic diversity in TLC. For these lineage B populations, the nucleotide and haplotype diversities in mainland China populations (e.g., SRC and JRC populations) were higher than the values from the other populations (e.g., SMR and HKR), and this supported the conclusion that the oriental river prawn originated in mainland China and dispersed to other areas.

Although two lineages were found (Figs 1, 2 and 3), not all ten populations simultaneously included both individuals from lineages A and B. The individuals in SMR, SRC and HRK were only found to belong to lineage B, and the specimens from MTR, TLC, MRC and JRC were included in lineage A or B, respectively, while the specimens in CLR, BLJ and KLJ were only discovered in lineage A. This may partly result from different dispersal routes and different times of origination for the two lineages, and their arrival time was apparently less than one million years ago [11, 12, 43]. Demographic expansions were observed in each lineage, starting in the second-to-latest interglacial period for lineage A (209,533 yrs ago) and within the last glacial period in lineage B (49,248 yrs ago). The event of connecting the islands of Taiwan, Japan and Korea to the mainland China occurred 2 to 3 times in the Pleistocene [44, 45, 46]. Our pervious paper [16] supported that (1) lineage A was older than lineage B, (2) lineages A and B may originate from the same ancestor in mainland China and were then dispersed to Taiwan at different times, and (3) lineage A moved to Taiwan earlier than lineage B.

For lineage B, the most common allele was shared by all populations (HRK, SMR, MRC, JRC and SRC populations), and this indicated all populations have the same ancestor. Populations with ancestral genotypes tend to preserve higher nucleotide and haplotype diversities because of the long-term accumulation of mutations [40, 41, 42]. The nucleotide diversity (π = 0.009) and haplotype diversity (h = 0.867) in SRC population was significantly higher than the values from the other populations, and the HRK population had the lowest nucleotide and haplotype diversities (Table 1). Therefore, the SRC and HRK populations were the oldest and the youngest populations, respectively. Furthermore, the oriental river prawn originated in mainland China [12]. Based on above discussions, the dispersal route of lineage B might be from the south of China (SRC), north to higher latitude areas (MRC, JRC and Taiwan), and further north to the north of China (TLC) and Korea. For lineage A, we also found that KLJ and BLJ populations located the most north sampling areas liked as HRK population in lineage B had the lowest nucleotide and haplotype diversities. Furthermore, the relationship between KLJ (located in the eastern Japan) and TLC (in the north of China) populations was not such close as the one between BLJ (located in the western Japan) and TLC (Fig 3). Thus, two different dispersal routes might yield for lineage A. The first route is from the south of China, north to higher latitude areas (MRC, JRC and Taiwan), and further north to eastern Japan (KLJ); and the other one is from the south of China, north to higher latitude areas (MRC, JRC and Taiwan), north to the north of China (TLC), and to western Japan (BLJ).

Significant genetic differences were found in most pairs of the ten populations (Table 2), and the hierarchical AMOVA revealed that a significant genetic structure across all hierarchical levels existed among the ten populations, but no obvious geographic division was found in genealogic reconstructions except for TLC in lineage A (Figs 2 and 3). This outcome is different from the outcomes that were often found of a high degree of phylogeographically related genetic structure [11, 42, 47, 48, 49, 50, 51, 52, 53, 54]. This might be because the expansion time for lineage A (209,533 yrs ago) or lineage B (49,248 yrs ago) were too short to accumulate enough genetic variation and form geographically unique clade [55, 56, 57]. The haplotypes of lineage A_2_ (TLC population) were close to BLJ haplotypes, and this might result in the secondary back contact from BLJ populations.

There was no significant genetic divergence between MRC and JRC, which may result from frequent gene flow because the geographical distance between JRC and MRC is very close and their environments were similar (Fig 1). The transportation of *M*. *nipponense* from SRC to SMR for aquaculture or the maintenance of genetic diversity [54] may partly explain the lack of genetic variation between SRC and SMR populations.

## Conclusions

Our study indicated a high level of genetic structure among the oriental river prawn populations in East Asia. Two main lineages (A and B) were found, and lineage A was older than lineage B. A rough estimate of the ages of lineages A and B were obtained and were approximately 490,000 and 253,000 years old, respectively. Lineages A, A_1_, A_2_ and B of the oriental river prawn *M*. *nipponense* have experienced population expansion since the Pleistocene glacial cycles in East Asia (approximately 209,533, 188,371, 36,100 and 49,248 yrs ago, respectively). The possible dispersal routes of *M*. *nipponense* in the East Asia were also speculated. Lineage B could have experienced expansion more recently than lineage A. The phylogeography and genetic structure of *M*. *nipponense* in East Asia might be influenced by land bridges during Pleistocene glacial maximums and by human colonization.

## Supporting information

S1 Dataset All sequences of 16S rRNA. (FASTA)

S2 Dataset All sequences of COI. (FASTA)

S3 Dataset All combined sequences of 16S rRNA and COI. (FASTA)
